# ACRODERMATITIS ENTEROPATHICA: CLINICAL MANIFESTATIONS AND PEDIATRIC DIAGNOSIS

**DOI:** 10.1590/1984-0462/;2018;36;2;00010

**Published:** 2018-01-15

**Authors:** Ieda Regina Lopes Del Ciampo, Regina Sawamura, Luiz Antonio Del Ciampo, Maria Inez Machado Fernandes

**Affiliations:** a Universidade Federal de São Carlos, São Carlos, São Paulo, Brasil.; b Universidade de São Paulo, Ribeirão Preto, São Paulo, Brasil.

**Keywords:** Zinc deficiency, Zinc, Child, Deficiência de zinco, Zinco, Criança

## Abstract

**Objective:**

To report a case of acrodermatitis enteropathica, a rare disease with autosomal recessive inheritance.

**Case description:**

An 11-month-old boy was presenting symmetrical erythematous and yellowish-brownish crusted lesions on his face, feet, hands and knees, intermittent diarrhea, fever, and recurrent infections since the age of six months. He was thin and had scarce hair on the scalp. The serum zinc level was measured and a reduced level of 27.0 mcg/dL (normal range: 50.0-120.0) was identified. Oral supplementation with 2.0 mg/kg/day of zinc sulfate was immediately initiated. A rapid and progressive improvement of symptoms was observed. The symptoms reappeared with an attempt to stop supplementation.

**Comments:**

Recognizing and properly treating acrodermatitis enteropathica is important to prevent complications.

## RESUMO

**Objetivo:**

Relatar um caso de acrodermatite enteropática, doença rara com herança autossômica recessiva.

**Relato de caso:**

Menino de 11 meses de idade apresentava lesões eritematosas simétricas na face, pés, mãos e joelhos, diarreia intermitente, febre e infecções recorrentes desde os seis meses de idade. Ele estava emagrecido e tinha cabelos escassos no couro cabeludo. Foi solicitada a avaliação da concentração sérica de zinco e identificado um nível reduzido de 27,0 mcg/dL (valores de referência: 50,0-120,0). Foi iniciada a suplementação oral com 2,0 mg/kg/dia de sulfato de zinco. Houve melhora rápida e progressiva dos sintomas. Os sintomas reapareceram quando houve uma tentativa de descontinuar a suplementação.

**Comentários:**

Reconhecer e tratar adequadamente a acrodermatite enteropática é importante para evitar as complicações da doença.

## INTRODUCTION

Acrodermatitis enteropathica is a rare and severe genetic disorder, of autosomal recessive inheritance, which determines the deficiency of the intestinal absorption of zinc, an essential trace element required by more than one hundred enzymes and whose role in the metabolism of nucleic acid is important.^1^ ^,^ ^2^ The gene *SLC39A4*, located in chromosome 8q24.3, codifies the transmembrane protein required for zinc absorption (Zip4), which is expressed in the duodenum and jejunum, and its mutation reduces the intestinal ability to absorb dietetic zinc.^3^ ^,^ ^4^

In Brazil, the frequency of this condition is unknown, but the estimation is that 1.5 million people are affected by it. There is no preference for sex or race, and it typically occurs at early ages, little after the conclusion of breastfeeding, with posterior introduction of cow milk in the diet.^5^

The classic clinical manifestations of acrodermatitis enteropathica are characterized by the triad eczematous and erosive dermatitis, acral and perioficial symmetrical dermatitis, alopecia and diarrhea. Paronychia, onicodistrophy, angular stomatitis, cheilitis, conjunctivitis and photophobia can also take place.^6^ ^,^ ^7^ The disorder progresses with difficulties regarding: weight gain, delayed growth, neuro-psychic disorders, delayed puberty, male hypogonadism, anemia, anorexia, hipogeusia and difficulty to heal wounds.^5^ Besides, whatever the cause is, zinc deficiency changes immunity, contributing with the high predisposition to fungal and bacterial infection, which can trigger systemic severe scenarios, and with the high mortality rates in developing countries.^8^ ^,^ ^9^

The gold standard for the diagnosis of acrodermatitis enteropathica is plasma zinc deficiency, which, however, may present serum concentrations within normal reference patterns, even when there is tissue depletion. Therefore, the dosage of alkaline phosphatase can be useful. Since it is a zinc-dependent enzyme, it responds to its replacement by increasing the low serum levels observed initially.^10^

Maternal milk functions as a protective food, since it contains zinc ligands, which contribute with its absorption, thus masking the deficiency inherited from the proteins that transport this micronutrient. On the other hand, children who are breastfed with milk poor in zinc - rare condition - may present with a transient newborn deficiency of this micronutrient, with clinical manifestations similar to those of acrodermatitis enteropathica. In this case, it is curious that the maternal supplementation of zinc does not improve the quality of the human milk. So, the children who are breastfed by these mothers should be kept on zinc supplementation until weaning, which can afterwards be suspended, since they present with normal zinc absorption.^2^

Other causes predispose to zinc deficiency and should be considered for the differential diagnosis of acrodermatitis enteropathica (Chart 1). The proper diagnosis is important to avoid the unnecessary zinc supplementation, which may lead to copper deficiency and immunological malfunction.^11^ ^,^ ^12^ ^,^ ^13^

**Chart 1:** Diseases to be considered for the differential diagnosis of dermatites enteropathica.

Acquired zinc deficiency
Biotin deficiency and multiple carboxylase deficiencies
Malabsorption syndromes secondary to cystic fibrosis or intestinal diseases
Essential fatty acid deficiency
Kwashiorkor
Acquired immunodeficiency syndrome
Isoleucine deficiency in restrictive diets for the maple syrup disease or methylmalonic aciduria
Glutaric aciduria type 1
Leucinosis
Nonketotic hyperglycinemia
Prematurity
Inadequate supplementation in parenteral feeding
Atypical epidermolysis bullosa
Atopic dermatitis
Cutaneous candidiasis
Seborrheic dermatitis

Source: adapted from Gehring KA. Curr Opin in Pediatr. 2010;22:107-12.

Since this condition is rare and due to the importance of its early diagnosis, the objective of this publication was to describe aspects of acrodermatitis enteropathica using a case report of a pediatric patient affected by the disease.

## CASE REPORT

Male child, born of natural delivery at term, without intercurrences. Only child of a nonconsanguineous couple, without family history of acrodermatitis enteropathica, was reffered to the service at the age of 11 months, with history of skin lesions since the age of six months, when the diarrhea also began. A few days after these manifestations, he had been diagnosed with “throat infection”, and, therefore, was treated with penicillin benzathine. Ten days after the use of the antibiotic, there was a reddish lesion on the corner of the eye, which then developed to the feed, hands, knees, gluteus, and perioral region, with diagnostic hypothesis of pharmacodermia. However, from the seventh to the eleventh month of life, the cutaneous lesions persisted, and the intermittent fever and diarrhea accompanied the clinical chart, requiring two hospitalizations. The patient was fed with breastmilk, complemented with formula based on cow’s milk protein, from birth to the age of six months, when both were suspended, and whole powdered cow’s milk was introduced.

**Figure 1:**
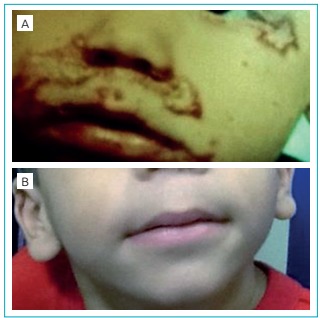
Patient at the age of seven months (1A) and two years (1B).

In the physical examination he was thin, weighing 6,920 g (Z score= 1.95), measuring 60.5 cm (Z score = 4.86), with body mass index (BMI)=18.9 (Z score = 1.48), sparse and whitened hair, perineal erythematous lesion and exulterated lesions with mild desquamation and meliceric crust on the face, feet, hands and knees, besides whitened lesions on the mouth (Figure 1). The doses of the serum concentrations revealed: zinc=27.0 ­mcg/­dL (50.0-120.0); total protein=6.0 ­g/­dL (6.0-8.5); albumin=4.0 ­g/­dL (3.8-5.4); alkaline phosphatase=127.0 U/L (65.0-300.0); serum iron=60.0 ­mcg/­dL ­(­40.0­-­160.0); immunoglobulins IgG=447.0 mg/dL (350-1180), IgM=62.6 mg/dL (36-104) and IgA=38.9 mg/dL (36-165); glycemia=71.0 mg/dL (70-100); and negative antiendomysial antibody (IgA). The xylose absorption test was 52.0 mg% (reference value>25,0); fecal steatocrit, 11% (reference value≤2%); and the sweat chloride test, 11.8 mEq/L (reference value ≤40), that is, except for the zinc, they were all normal.

With the diagnostic hypothesis of acrodermatitis enteropathica, the oral replacement with 2.0 mg/kg/day of zink sulfate began. There was fast and progressive improvement of the lesions and in the nutritional status, and the child no longer presented with diarrhea or fever. A few months after the improvement of the symptoms and the recovery of the nutritional status, family members interrupted the prescribed zinc on their own, and at that moment the diarrhea and the skin lesions returned. The treatment was immediately reestablished.

At the age of two years and eight months, the serum zinc concentration was equal to 58 mcg/dL. The child remained without skin lesions (Figure 1), weighing 15,650 g (Z score=134), height of 87.5 cm (Z score=1.49) and BMI=20.7 (Z score=3.19).

## DISCUSSION

For five months, the patient presented with the clinical manifestations of zinc deficiency, such as intermittent diarrhea, growth delay, dermatitis and alopecia, which suggested a clinical picture characteristic of acrodermatitis enteropathica. Corroborating the clinical manifestations, serum zinc values below normal were also observed, which is common in the classical type of the disease. However, it is important to mention that normal serum levels of zinc have been observed in 30% of the cases in general, which does not rule out the disease.^5^ Besides, since zinc is transported by albumin, serum levels below normality can be found in some conditions associated with hypoalbuminemia, such as protein-energy malnutrition.

To consider the diagnosis of primary acrodermatitis enteropathica, it is important to rule out acquired zinc deficiency of any etiology, such as: insufficient zinc supply by parenteral feeding; inadequate zinc stocks due to premature birth; poor absorption due to cystic fibrosis or small intestine resection; acquired immunodeficiency syndrome (AIDS); atypical epidermolysis bullosa; generalized or local candidiasis; abnormal metabolism of essential fatty acids; seborrheic dermatitis; kwashiorkor; iatrogenic isoleucine deficiency due to restrictive diets for the maple syrup urine disease; and methylmalonic aciduria or phenylketonuria. Recently, two cases of food allergy were described with serious zinc deficiency.^14^

Since acquired zinc deficiencies should be part of the differential diagnosis, the specific tests for this purpose were conducted during the initial evaluation of the patient, and were within normality ranges, which led to the ruling out of the initial hypothesis of poor intestinal absorption, including celiac disease, AIDS and cystic fibrosis. The other diagnoses were ruled out due to the substantial improvement of the patient after zinc supplementation, for example, the hypothesis of food allergy, since improvement was observed without the exclusion of cow’s milk. The family tested the efficacy of the supplementation after spontaneously stopping the zinc therapy. This led to the reappearance of the signs and symptoms, and the posterior clinical improvement after its reintroduction.

Since this is an autosomal recessive disease, the detection of the mutation in gene *SLC39A4*, located in band 8q24.3, could have helped to diagnose acrodermatitis enteropathica. However, this test still has high costs to be introduced in the clinical practice routine.

The treatment of acrodermatitis enteropathica consists on the supplementation of oral zinc, and the recommendation is of one dose between 1 and 3 mg/kg/day of elemental zinc.^5^ Zinc sulfate has been described as the most tolerated compound, even though it can be administered as acetate and gluconate. In the case described, the adequate clinical response to oral zinc supplementation and the recrudescence of signs and symptoms to the removal of its supplementation are in accordance with studies that reveal the need of these two factors for the diagnostic confirmation of the disease.^15^

The case described corroborates the importance of including congenital acrodermatitis enteropathica in the differential diagnosis of clinical cases of children presenting with acral and perioficial dermatitis, diarrhea, recurrent infections and alopecia.
